# Skill-Related Adaptive Modifications of Gaze Stabilization in Elite and Non-Elite Athletes

**DOI:** 10.3389/fspor.2022.824990

**Published:** 2022-04-12

**Authors:** Susanne M. van der Veen, Alexander Stamenkovic, James S. Thomas, Peter E. Pidcoe

**Affiliations:** ^1^Department of Physical Therapy, Virginia Commonwealth University, Richmond, VA, United States; ^2^Department of Physical Medicine and Rehabilitation, Virginia Commonwealth University, Richmond, VA, United States; ^3^Department of Biomedical Engineering, Virginia Commonwealth University, Richmond, VA, United States

**Keywords:** vestibular ocular reflex, gymnastics, cancellation, suppression, training, gaze, visuomotor

## Abstract

The vestibular ocular reflex (VOR) provides gaze stability during head movements by driving eye movements in a direction opposing head motion. Although vestibular-based rehabilitation strategies are available, it is still unclear whether VOR can be modulated by training. By examining adaptations in gaze stabilization mechanisms in a population with distinct visuomotor requirements for task success (i.e., gymnasts), this study was designed to determine whether experience level (as a proxy of training potential) was associated with gaze stabilization modifications during fixed target (VOR promoting) and fixed-to-head-movement target (VOR suppressing) tasks. Thirteen gymnasts of different skill levels participated in VOR and VOR suppression tasks. The gain between head and eye movements was calculated and compared between skill levels using an analysis of covariance. Across experience levels, there was a similar degradation in VOR gain away from −1 at higher movement speeds. However, during the suppression tasks, more experienced participants were able to maintain VOR gain closer to 0 across movement speeds, whereas novice participants showed greater variability in task execution regardless of movement speed. Changes in adaptive modifications to gaze stability associated with experience level suggest that the mechanisms impacting gaze stabilization can be manipulated through training.

## Introduction

Experienced coaches who train athletes to perform aerial acrobatics skills (i.e., gymnastics, diving) have long observed the importance of visual cues (Hondzinski, 1998; Davlin et al., 2004). The regulation of flight time and an estimation of landing (or time of collision T_c_) are the key factors. Full vision improves landing success, and a prospective control strategy uses not only take-off parameters in a feedforward manner, but also vision as feedback to determine landing time (Bardy and Laurent, 1998). Novice athletes sometimes ignore or are unaware of these cues and rely on timing to complete aerial acrobatics successfully. This is considered an open-loop control strategy and is often used in lower-level skill execution. However, when they start to do more advanced aerial activities, visualization of the landing area becomes increasingly important to confirm a safe and stable landing (Davlin et al., 2004). Flipping (lower level) skills are defined as aerial rotations around a medial–lateral axis. When twisting (higher level) is added to these aerial activities (rotation around a superior–inferior axis), visualizing the landing zone becomes increasingly complicated since head motion is now multi-axial. Vestibular information about the orientation of the head relative to the ground is lost during flight (Bardy and Laurent, 1998). Experience and training not only improve the development of gaze stabilization, but also promote skill progression and the successful execution of higher-level, multiple rotation, and multi-axis activities. This paper describes the relationship between training and the ability to manipulate gaze stabilizing eye movements that are counter to the gain of the vestibular ocular reflex (VOR).

The VOR facilitates a stable visual field and reduces retinal smear in tasks that necessitate head motion by driving eye movements in a direction opposite to those of the head (Baloh et al., 1984). However, when executing aerial acrobatics skills requiring multi-axis rotations that include both active and passive had motions, the action of the VOR may produce performance decrements since the landing surface may be outside the field-of-view (FOV) several times during skill performance. Specifically, when the landing surface is outside the FOV (behind or below the athlete), the VOR would delay the ability to see this surface since eye gaze would be driven in a direction opposite to the twist (away from the prospective landing surface). In this paper, the term VOR supression (VORs) is used to describe an eye movement that is counter to the normal VOR. VORs would bring that surface into the FOV sooner and facilitate a faster acquisition of the area of interest as it comes into view. This circumstance would promote gaze toward the landing site and may provide a landing time estimate to the participant. The ability to modify gaze stability has been found to occur in populations who are frequently exposed to twists and aerial acrobatics (e.g., dancers, ice-skaters, and pilots during specific tasks) (Lee et al., 2004; Alpini et al., 2009; Maheu et al., 2019). The presence of context-specific control of VOR gain provides evidence that VOR can be manipulated with exposure and training. How VORs is promoted with graded exposure to multi-axial twisting and flipping (i.e., gymnastics) as a function of skill level is unknown. Higher skill level athletes are typically exposed to activities that require higher angular velocities to complete successfully. The presence of context-specific control of VORs in this population may provide evidence that VOR gain can be manipulated with exposure and training.

**Figure 1:**
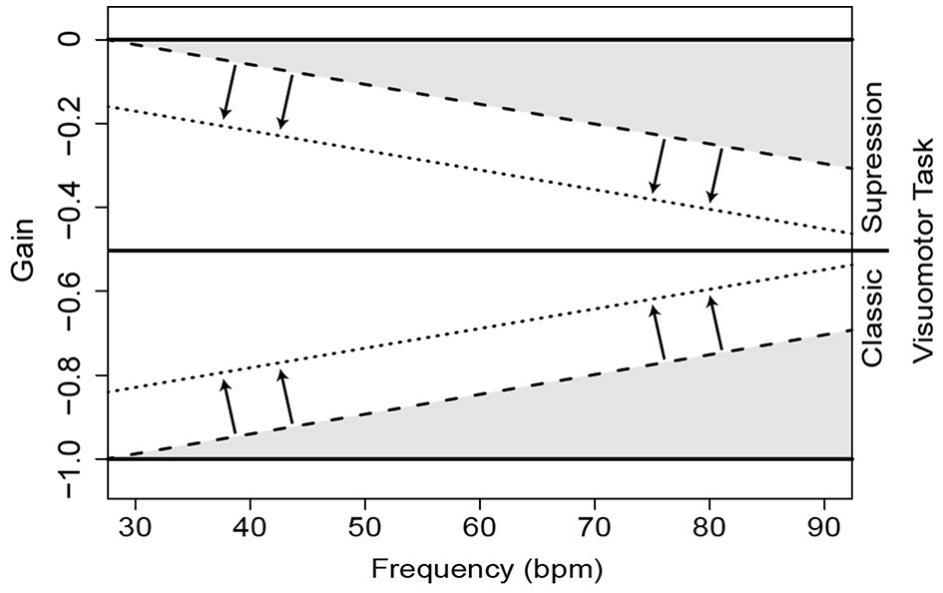
Prediction schema of VOR gain relationship changes as a function of experience level and movement speed. Solid lines represent an optimal performance across tasks as movement speeds increase for the VOR suppression (top) and classic visuomotor task (see bottom). Decrements in performance due to experience could occur through rotation of the regression line (e.g., dashed lines and shaded portions), with steeper slopes indicating poorer performance, or through translation of the regression line (e.g., dotted lines, see arrows), representing a shift in baseline performance.

In an effort to understand how exposure and training affect the adaptive modification of gaze stability, this exploratory study investigates how VOR gain changes during classic and suppression-based visuomotor tasks at 7 different head velocities in athletes trained to perform multi-axial airborne rotational skills. Specifically, we predict that there is a positive correlation between an athlete's skill level and VOR gain (i.e., as experience level decreases, associations between VOR gain and head velocity would deviate from the optimal values of −1 for classic visuomotor task and 0 for VOR suppression visuomotor task) as evidenced by either a rotation (Figure 1—shaded) or translation of the regression line of this relationship (Figure 1—arrows).

## Methods

### Participants

In this study, gymnasts with a skill level of 5 have limited training or exposure to aerial skills. They act as a control group comparison. At Level 6, uniaxial aerial skill is introduced. As gymnasts progress to Level 10, the complexity of the aerial skills increases and these athletes are required to complete 3 different kinds of flips and an acrobatic series with at least two connected flips.

Thirteen female gymnasts (21.0 ± 5.7 year) took part in this preliminary study (Table 1). Their ability level ranged from novice to elite with seven participants of the 13 still active in competition. They were consented on-site and told that they could exit the study at any time without recourse. The study was approved by the Internal Review Board of Virginia Commonwealth University (IRB Approval HM2M0008030). Age, preferred twist direction, hand dominance, and level of competition were acquired *via* self-report.

**Table 1:** Participant demographics.

**Subject**	**Age**	**Twist direction**	**Hand dominance**	**JO** **level**
01	16	R	R	10
02	20	R	R	10
03	14	R	R	8
04	16	R	R	8
05	16	R	R	10
06	18	L	R	5
07	14	L	R	8
08	13	L	R	7
09	13	L	R	7
10	15	L	R	5
11	27	L	R	8
12	23	L	R	9
13	26	R	R	9

*Level represents competition level based on USA Gymnastics Junior Olympic (JO) Program, with higher numbers indicating a more advanced gymnast*.

### Experimental Setup

All data were collected using a kinematic system to monitor head and trunk position with 6 degrees-of-freedom and a binocular eye dark-pupil tracking system to monitor horizontal and vertical eye position. Data collection with these two systems was integrated providing a synchronous dataset. The participants were seated on a wooden stool at the end of a 0.76-m-high workbench. They were asked to wear a helmet fitted with a camera-based infrared eye-tracker (EyeLink II, SR Research Ltd. Mississauga, Ontario Canada). The eye-tracker had a tracking range of ± 30° in the horizontal direction and ± 20° in the vertical direction with resolution of 0.01°. These data were collected at 250 Hz and provided eye-in-head position. Head and trunk position were collected from two electromagnetic (EM) motion sensors (Motion Monitor™, Innovative Sports Training, Chicago, Illinois, USA). One EM sensor was on the helmet that also held the eye-tracking hardware and provided head-in-space position. The second was mounted on a strap worn around the participant's thorax providing a head-on-trunk position. Data were collected from the kinematic system at 100 Hz.

A transmitter was located behind the subject and provided orthogonally oriented electromagnetic fields. The theory of operation for this type of sensor is that it orients itself based on transmitter field strength. The field strength decreases as a square of the distance from the transmitter. The transmitter used in this application had a functional radius of 3 m. The participants (and sensors) were located well within this range. Care was taken to avoid the presence of metal within the transmitter field to minimize eddy current distortions. To further reduce any effect of metal, a mapping procedure was performed prior to data collection. This metal mapping procedure uses known sensor location data to construct a distortion map of the collection space. These data are then used to linearize any measurement error across the mapped space with a resolution of 0.5 mm linearly and 0.1° angularly. All collected data were stored in a coded file using subject initials, the date, and trial number or descriptor.

### Participant Setup

After securing the helmet and sensors, the subject was asked to sit steady and face an LED display placed on the workbench at a distance of 1 m. The subject was positioned at eye level with the center target on this board. A world-based right-hand coordinate system was defined on the workbench top. The origin was located to the participant's right. Subject landmarks were defined in a prescribed manner using a stylus fixed to a free-floating sensor. These standardized locations allowed the reconstruction of a rigid body model of the subject for interpretation of position data. Left–right symmetry was assumed.

### Visuomotor Task

#### Classic Condition: Static Target Fixation (VOR)

**Figure 2:**
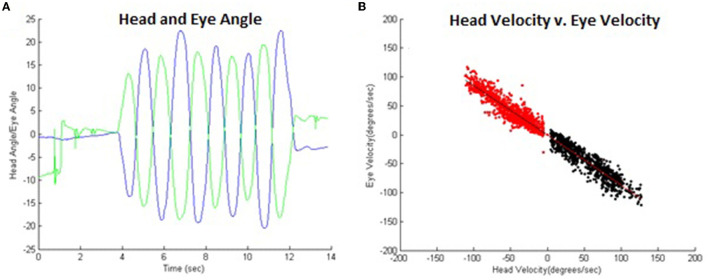
**(A)** Typical example of VOR temporal head (blue) and eye (green) angle data. **(B)** These same data comparing head vs. eye angular velocity illustrating VOR. Leftward (black) and rightward head movements (red) are separated for analysis with head velocities below 5°/s excluded since the VOR response is negligible at low head velocities. The VOR gain was ~-0.8 in this example.

In the classic visuomotor condition, participants were seated at the end of a cloth-draped tunnel to remove the visual scene and visually fixated on a single LED target located centrally on the calibration screen and subtended to a visual angle of 0.03°. Participants were then instructed to actively rotate their head in the transverse plane (yaw or “no” motion) while continuing to fixate on the LED target for an approximate movement amplitude of ± 15° from neutral head position. Active head rotation was selected for this experiment since it mimicked the *in situ* performance environment of the gymnast. Movement speeds were dictated by a metronome set at seven different frequencies ranging from 72 to 196 beats/min with increments of 20 beats/min. This combination of speed and amplitude resulted in angular movement velocities between 35° and 100°/s. Participants were verbally encouraged to maintain the prescribed angular head range during the higher tempos to provide a full range of angular head velocities. Following each trial of 5 complete rotation cycles, head range of motion was reviewed, and the trial repeated if necessary. A typical example of these data is illustrated in Figure 2A. Angular velocities of the head (from the head-mounted sensor) and eye were calculated using a Savitzky–Golay differentiation filter. After removing any stationary head movement data first (<3° amplitude), VOR gain was calculated *via* a custom MATLAB script as the average of a point-by-point ratio between head and eye velocities (Figure 2B).

#### VOR Suppression Condition: Fixation of Head-Mounted Target

**Figure 3:**
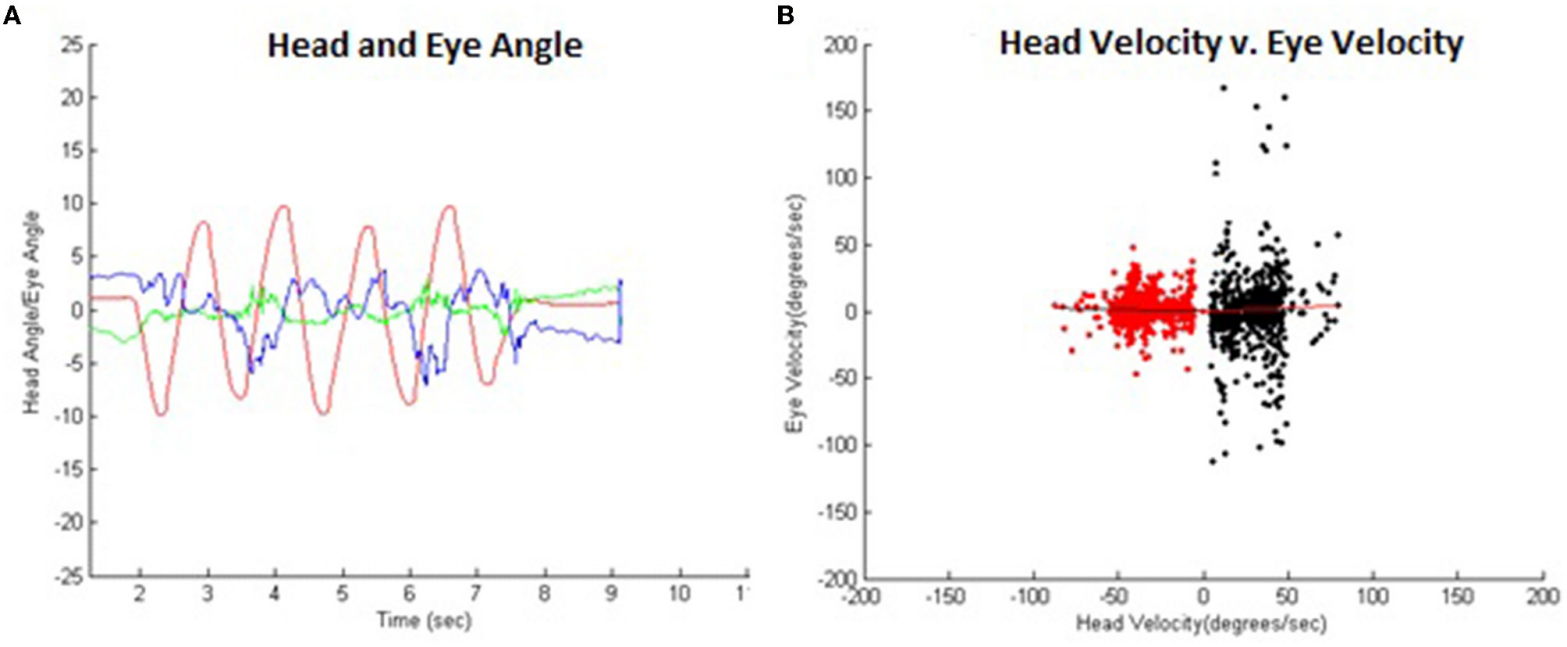
**(A)** Typical example of VOR suppression temporal head (red), right eye (blue), and left eye (green) angle data. **(B)** These same data comparing head vs. eye angular velocity illustrating VOR suppression. Leftward (black) and rightward head movements (red) are separated for analysis with head velocities below 5°/s excluded since the VOR response is negligible at low head velocities. The VOR gain was approximately zero in this example.

In the VOR suppression condition, the calibration display was replaced with a black trifold foam board. A laser pointer was fitted on the participant's helmet, so the red dot appeared at the center of the trifold foam. This configuration fixed the laser generated target to head motion. The target subtended a visual angle of ~0.01°. Participants then repeated the same movement amplitude and speed conditions of the classic condition described above, while maintaining stable gaze at the target created by the head-fixed laser pointer. A typical example of these data is illustrated in Figure 3 for position (Figure 3A) and angular velocity (Figure 3B) for the head and eye.

### Data Analysis

Horizontal eye and head angular data were used to compute the VOR gain. Any data offset was removed by subtracting the mean of 500 ms of pretrial non-movement initial data from the entire trial. Left and right head movement data were separated for analysis. Trials missing data were removed from the analysis, 3 trials for the VOR condition within one participant (13) due to failing to maintaining the angular velocity at the higher metronome speeds, and 6 VOR suppression trials for 2 participants (13 and 01) due to difficulty maintaining metronome speed (13), or eye tracking errors (01). In total, a remainder of 88 VOR and 85 VOR suppression trials were analyzed. Task initiation and termination were defined as the first and last instance the absolute head angle exceeded 3° to remove static eye and head data from the trial (represented in Figures 2B, 3B as the band around reflecting turning points of head motion). The range of head movement was calculated as the average amplitude of the head oscillation (VOR 29.1 ± 8.4°, VOR suppression 29.9 ± 7.3°). The frequency of the movements was calculated as the number of oscillations made in the time between task initiation and termination. Angular velocity was calculated by determining the time rate of change of the head and eye angular position data. As mentioned previously, VOR gain was calculated *via* a custom MATLAB script as the average of a point-by-point ratio between head and eye velocities.

### Statistical Analysis

Correlations for repeated measures were undertaken using the rmcorr package in R (version 0.4.1, Bakdash and Marusich, 2017). Briefly, this method accounts for non-independence among observations using analysis of covariance (ANCOVA) to statistically adjust for interindividual variability. Correlation coefficients accounting for repeated measures (r_rm_) are presented, with 95% confidence intervals estimated *via* bootstrapping (number of iterations = 1,000).

To test whether the relationship between VOR gain and movement frequency changed as a function of experience level, significance testing was undertaken on regression coefficients (i.e., slope). Coefficients were first normalized using Fisher's z-transformation, with the difference between z-transformed coefficients compared with a critical z-score (Suzuki et al., 2008; Weaver and Wuensch, 2013). To account for multiple comparisons, Bonferroni adjustments were applied (such that critical Z = 2.58, *p* < 0.005).

## Results

### Angular Head Velocity

#### Classic Condition: Stationary Visual Target

**Figure 4:**
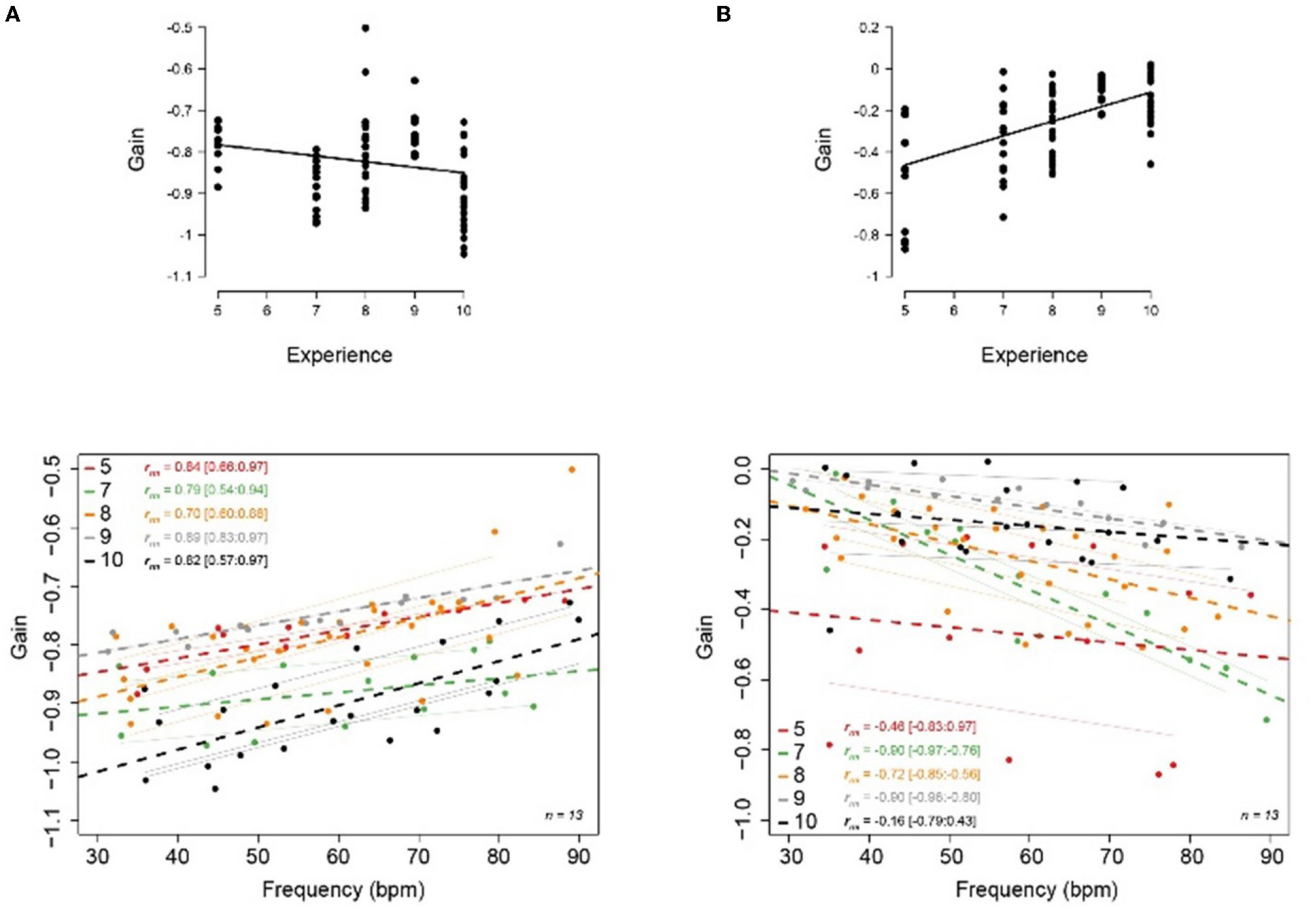
Regression lines comparing head frequency with VOR gain during head rotation for the different skill levels (colored) and participants (light gray) for the **(A)** classic condition and **(B)** VOR suppression condition. The top panels represent group average, over all participants and the different velocities, the bottom panels represent gain regression lines for the groups (in color) and per gymnast (gray).

Associations between VOR gain and experience levels across all movement speeds showed few changes in task accuracy as experience level increased (Figure 4A, top panel) despite clear changes in the proportion of trials closer to optimal for the most elite and novice groups (i.e., Level 10 vs. Level 5). When examined on a participant basis (Figure 4A, bottom panel), VOR gain and movement speed showed a strong positive relationship (*p* < 0.001) regardless of experience level with intercept values close to −1 (−1.12 to −0.88) with a positive slope (0.0013–0.0038), which indicates relative deterioration in task execution as movement speeds increased.

#### VOR Suppression: Head-Fixed Target

In contrast to the classic visuomotor task, VOR gain showed a significant negative relationship to frequency (*p* < 0.001), with, greater changes as a function of experience level in the VOR suppression task (Figure 4B, top panel). This could be largely attributed to the greater variability and poorer execution shown by novice participants (e.g., Level 5 values deviating from 0). When compared to movement speed (Figure 4B, bottom panel), higher experience levels (i.e., Levels 9 and 10) showed a greater maintenance of successful VOR suppression even with increasing movement speed, with regression coefficients (i.e., slope) and y-intercepts nearing zero (e.g., Level 10, regression coefficient = −0.0008, y-intercept = −0.09). This contrasted with novice participants, who showed a general inability to successfully suppress VOR across the range of movement speeds (as indicated by a shift in the regression line and deviation of y-intercept away from 0, i.e., Level 5 = −0.26). Despite the magnitude in changes between regression coefficient values between experience levels, statistical analysis showed that there was insufficient evidence to confirm such differences. In contrast, significant differences found between experience level in VOR suppression intercepts and slopes were seen between Level 5 vs. Level 7 (*p* < 0.001) and Level 8 vs. Level 9 (*p* < 0.001).

## Discussion

This study investigated whether gymnasts of differing experience levels exhibited adaptations in VOR gain that reflected task requirements. VOR gain showed characteristic changes during the VOR suppression visuomotor task that tended to align with our expected predictions (refer to Figure 1), such that expert participants were able to maintain VOR gain values closer to zero compared to their novice counterparts, even at higher movement speeds. VOR suppression is a necessity for the aerial acrobatics part of the competition at these higher experience levels (i.e., Levels 9 and 10). VOR suppression, however, is likely to decline at higher speeds or in lower skill level gymnasts as exposure and demand of training are likely to have been less demanding of the skill to suppress VOR. Interestingly, VOR gain during the classic visuomotor did not show the same changes across experience level, which suggests that certain visuomotor task may have greater utility for training purposes.

These data showed that all gymnasts were able to achieve VOR gains near −1 at the low head velocities in the fixed visual target task; however, this relationship deteriorated at higher angular velocities. These results are similar to the general public and were expected, as VOR gains below an absolute 0.68 have been proposed as abnormally low (MacDougall et al., 2009). No statistical difference in VOR performance was found between gymnastics skill level. VOR suppression (or VORs), however, did show some statistical differences as a function of skill level. The higher skill level athletes had more stable VOR suppression gains (*r* = 0.019) and were able to maintain this VOR suppression (0.33 ± 0.48) throughout the range of imposed head angular velocities, as shown in the smaller slope. The lowest level athletes were more variable (r = 0.208) and showed a limited ability for VOR suppression (mean ± SD 0.17 ± 0.38) across head velocities. The intermediate skill levels showed a graded response in VOR suppression (0.22 ± 0.42) when compared to the expert and recreational athletes. This could be the reason why expert gymnasts are more reliant on visual information during salto (backward aerial summersaults) than novice gymnasts as described by Bardy and Laurent (1998).

Besides skill level, the ability to suppress VOR could be attributed to age as skill level and exposure to multi-axial twists increase with age in our population. However, evidence from student aviation pilots showed greater VOR gains (i.e., closer to −1 or 1) after training than students before training (Lee et al., 2004). Besides these results supporting the plasticity of the VOR, Lee et al. did not find any relationship between age and VOR. The development of VOR has shown to occur rapidly until the age of 6 years, when it then progresses at a slower rate to adult values by 16 years (Wiener-Vacher and Wiener, 2017). Although gymnast younger than 16 years of age were included in the experiment, VOR gains ranges for the gymnasts with ages below 16 were not different than the group older than 16 (−0.80 to 80 versus −0.71 to 0.87 respectively). Additionally, the younger gymnasts show to reach a VOR gain of −80 during the VOR suppression task, further indicating that age is not the driving factor in the ability to suppress VOR.

Because these athletes have a preferred twist direction, it was thought that the ability to suppress VOR would trend in that direction as well (i.e., VOR gain during leftward head motion would be closer to zero than during rightward head movement for the gymnasts that preferred leftward twists and *vice versa*). A secondary analysis showed that this was not supported by the data. No significant differences were found between left and right VOR gains for left and right head motion for either left or right preferred twisting gymnasts.

The correlation of VOR suppression to training dose suggests that VOR gain control can be enhanced through training. The training stimulus in this case is extreme and not directly suited for the treatment of patients suffering from concussion or traumatic brain injury (TBI). However, from a neurophysiologic standpoint, it may be important to alter VOR gain in certain circumstances to enhance visual input. Understanding this process in trained athletes has implications in the treatment of conditions that affect gaze stabilization ability. The gain of head and eye movements is disturbed resulting in retinal smear, which often causes symptoms of nausea and imbalance during movement (Crawford and Vilis, 1991; Paige, 1994; Gurley et al., 2013), for example, the cause of gait and balance problems following TBI and concussion (Parker et al., 2005, 2006; Kaufman et al., 2006; Pickett et al., 2007; Slobounov et al., 2008; Wares et al., 2015). The results of this study support the application of exposure and training to adapt the VOR to reduce these symptoms. However, the existing rehabilitation tools for training VOR gain are based on the qualitative estimates of head movement and gaze maintenance during treatment. In addition, patient reports of dizziness and nausea, often used to determine progression, can be problematic due to the variability of this metric relative to VOR gain. The lack of precision and standardization in evaluation and treatment of impairments in VOR gain has most likely contributed variability in treatment outcomes (Slobounov et al., 2008). Investigating how elite and novice athletes adapt their VOR may help us to understand how the integration of the sensory systems can be adapted in populations with poor balance. Therefore, this study will focus on VOR gain and VOR suppression in athletes trained to perform multi-axial airborne rotational skills.

## Limitations

Real plasticity and adaptability of the VOR can only be assessed in a longitudinal study, following VOR suppression ability in athletes such as gymnast's through their development and progress from recreational to elite status or follow a patient population with VOR deficits during the rehabilitation period. However, this study indicates gymnasts increase in the ability to suppress the VOR due to more elaborate exposure to airborne activities.

This is an exploratory study, and only 10 gymnasts were included in the recent study, which allows for 2 or 3 gymnasts per skill group. This is probably the cause of the limited significant results in this study. However, the trends and significant results present in the current work align with our initial prediction (see Figure 1) and reinforce the notion that mechanisms impacting gaze stabilization may be manipulated through training.

## Conclusion

The ability to suppress or cancel VOR increased with the level of experience in gymnastics and airborne skills. Therefore, it is likely that VOR can be trained or manipulated by exercises. However, further investigation is needed in the longitudinal development of VOR and VOR suppression in gymnasts and the effect of training populations with VOR deficits.

## Data Availability Statement

The raw data supporting the conclusions of this article will be made available by the authors, without undue reservation.

## Ethics Statement

The studies involving human participants were reviewed and approved by Internal Review Board of Virginia Commonwealth University. Written informed consent to participate in this study was provided by the participants' legal guardian/next of kin.

## Author Contributions

SV is responsible for the initial data analysis and manuscript preparation. AS is responsible for statistical and manuscript proof reading. JT and PP are responsible as laboratory managers and PI's on the project who proof read the manuscript.

## Conflict of Interest

The authors declare that the research was conducted in the absence of any commercial or financial relationships that could be construed as a potential conflict of interest.

## Publisher's Note

All claims expressed in this article are solely those of the authors and do not necessarily represent those of their affiliated organizations, or those of the publisher, the editors and the reviewers. Any product that may be evaluated in this article, or claim that may be made by its manufacturer, is not guaranteed or endorsed by the publisher.
